# Tibial slope in the posterolateral quadrant with and without ACL injury

**DOI:** 10.1007/s00402-021-04298-w

**Published:** 2021-12-28

**Authors:** A. Korthaus, M. Krause, G. Pagenstert, M. Warncke, F. Brembach, Karl-Heinz Frosch, J. P. Kolb

**Affiliations:** 1 Department of Trauma and Orthopaedic Surgery, University Medical Center Hamburg-Eppendorf, Martinistrasse 52, 20246 Hamburg, Germany; 2 Department of Diagnostic and Interventional Radiology and Nuclear Medicine, University Medical Center Hamburg-Eppendorf, Hamburg, Germany; 3 CLARAHOF Clinic of Orthopaedic Surgery, University of Basel, Basel, Switzerland; 4 Department of Trauma Surgery, Orthopaedics and Sports Traumatology, BG Hospital Hamburg, Hamburg, Germany

**Keywords:** Anterior cruciate ligament injury, Posterolateral tibial impression, Knee instability, Tibial slope, Standard value tibial slope

## Abstract

**Introduction:**

An increased tibial slope is a risk factor for rupture of the anterior cruciate ligament. In addition, a tibial bone bruise or posterior lateral impression associated with slope changes also poses chronic ligamentous instability of the knee joint associated with an anterior cruciate ligament (ACL) injury. In the majority of cases, the slope is measured in one plane X-ray in the lateral view. However, this does not sufficient represent the complex anatomy of the tibial plateau and especially for the posterolateral quadrant. Normal values from a “healthy” population are necessary to understand if stability of the knee joint is negatively affected by an increasing slope in the posterolateral area. Until now there are no data about the physiological slope in the posterolateral quadrant of the tibial plateau.

**Materials and methods:**

In 116 MRI scans of patients without ligamentous lesions and 116 MRI scans with an ACL rupture, tibial slope was retrospectively determined using the method described by Hudek et al. Measurements were made in the postero-latero-lateral (PLL) and postero-latero-central (PLC) segments using the 10-segment classification. In both segments, the osseous as well as the cartilaginous slope was measured. Measurements were performed by two independent surgeons.

**Results:**

In the group without ligamentous injury the mean bony PLL slope was 5.8° ± 4.8° and the cartilaginous PLL slope was 6.7° ± 4.8°. In the PLC segment the mean bony slope was 6.6° ± 5.0° and the cartilaginous slope was 9.4° ± 5.7°. In the cohort with ACL rupture, the bony and cartilaginous slope in both PLL and PCL were significantly higher (*P* < 0.001) than in the group without ACL injury (bony PLL 9.8° ± 4.8°, cartilage PLL 10.4° ± 4.7°, bony PLC 10.3° ± 4.8°, cartilage PLL 12.8° ± 4.3°). Measurements were performed independently by two experienced surgeons. There were good inter- (CI 87–98.7%) and good intraobserver (CI 85.8–99.6%) reliability.

**Conclusion:**

The bony and the cartilaginous slope in the posterolateral quadrant of the tibial plateau are different but not independent. Patients with an anterior cruciate ligament injury have a significantly steeper slope in the posterolateral quadrant compared to a healthy group. Our data indicate that this anatomic feature might be a risk factor for a primary ACL injury which has not been described yet.

**Level of evidence:**

III.

## Introduction

Anterior cruciate ligament ruptures due to a noncontact mechanism involve a force of ventral subluxation with valgus stress and internal rotation of the tibia [1, 2]. Within this mechanism, there is a possibility of femoral notch sign and impression fracture in the posterolateral quadrant of the tibial plateau due to the impact of the femur in the posterolateral region of the tibia [3]. In 49.3% out of 825 knees with an ACL tear Bernholt et al. detected an impaction of the lateral tibial plateau [4]. Multiple studies and the practical experience have shown that a posterolateral impression, which leads to a steeper slope is associated with a higher instability of the knee [5–7]. A relationship between ACL re-ruptures and a steep posterior tibial slope was found, as a greater tibial inclination should lead to a greater anterior tibial translation (ATT) when the knee joint is loaded [8–10]. The ACL is the major antagonist of ATT, so a higher tibial slope leads to an increased load on the ACL [7, 11]. A posterolateral impression leads to an increased anterolateral rotational instability and can results in a positive Pivot Shift test and a subjective instability [12, 13]. A remaining anterolateral instability results in a higher rate of re-ruptures after ACL reconstruction [14, 15]. Furthermore, it is well known that a steeper lateral slope is associated with posterolateral root tears in ACL injuries [13, 16]. Also, a steeper lateral slope was found to increase the risk of tibial tunnel widening [17].

The question that arises is: Do patients with an anterior cruciate ligament rupture have a steeper slope in the posterolateral quadrant than patients without an anterior cruciate ligament injury? And if so, what are the measured normal values on MRI for the corresponding segments?

Today there are no existing studies that define a “normal” value of the slope in the posterolateral quadrant of the tibial plateau. In order to decide whether a surgical reduction and for instance an arthroscopic guided screw fixation should be planned, it is crucial to define a norm value of the posterolateral tibial plateau [18]. In addition, the complex anatomy of the tibial plateau has to be considered. Therefore, a measurement of the tibial slope in just one sagittal plane, which is the standard X-ray is not sufficient [4, 5, 19]. The complex anatomy of the tibial plateau with a convex shape for the lateral plateau should be respected. The “Ten Segment-Classification” described by Krause et al. respects this anatomy [20]. Therefore, and for the first time, in this paper we describe the physiological slope in the posterolateral quadrant, which is divided into the posterolateral central (PLC) and posterolateral lateral (PLL) segment. We hypothesize that the tibial slope in the posterolateral Quadrant is steeper in the group with ACL injurie than in the healthy cohort.

## Methods

**Fig. 1:**
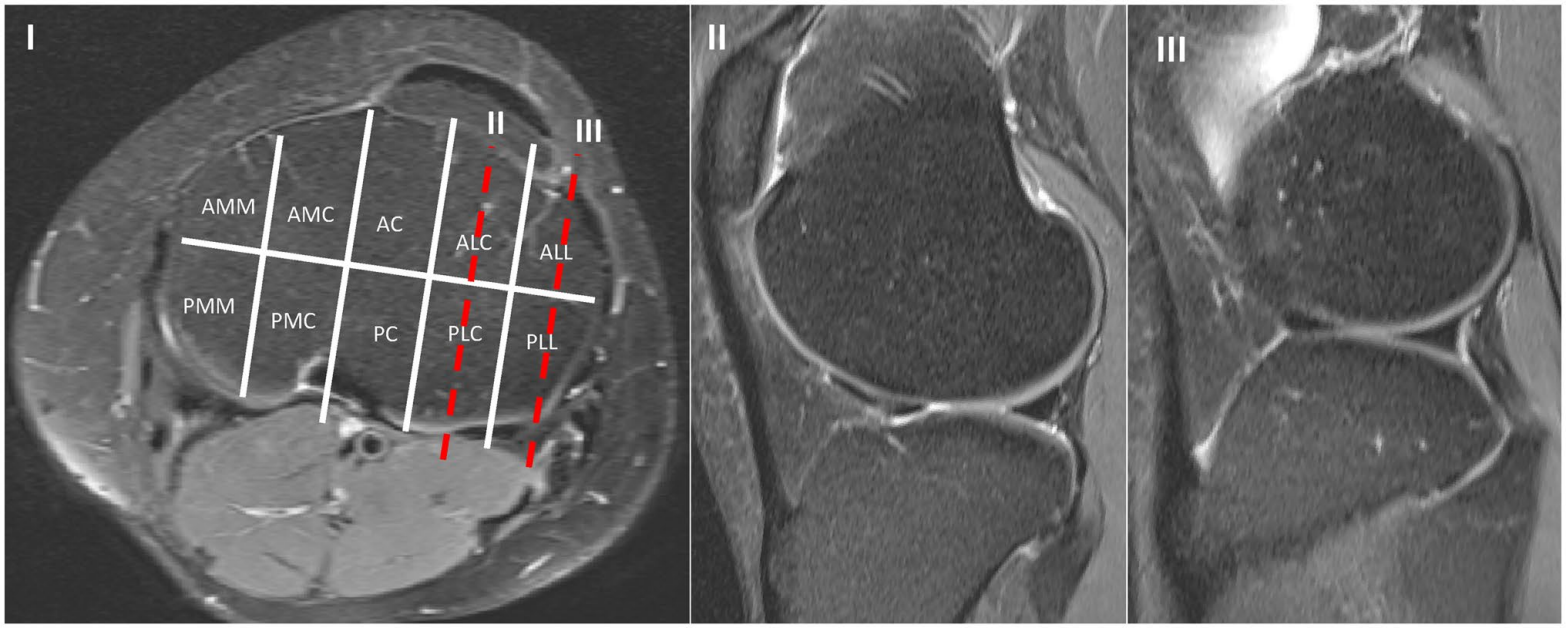
MRI of a 31 healthy woman. I axial slice of the MRI with applied 10 segment classification. The red dash line with the II shows the sagittal slice through the PLC segment, III shows the sagittal slice through the PLL segment. *ALL* antero latero-lateral, *ALC* antero-latero-central, *AC* antero-central, *AMC* antero-medio-central, *AMM* antero-medio-medial, *PLL* postero-latero-lateral, *PLC* postero-latero-central, *PC* postero-central, *PMC* postero-medio-central, *PMM* postero-medio medial; II sagittal section of the MRI's from I in the position of the red dash line marked II; III sagittal section of the MRI from I in the position of the red dashed line marked III

To plan the number of cases, we performed a power analysis with a 95% confidence interval and a width of 0.4. A collective size of 116 cases per group was determined. All knee joint MRIs performed at our center from 2018 to 2019 were examined until each of the groups was filled with 116 patients according to the inclusion criteria. In the group without ACL rupture, all MRIs were included that had no ligamentous injury and were older than 18 years. In the second group, all MRIs of the knee joint from patients over 18 years old with a confirmed ACL rupture were included. The examination was performed in the supine position in a 3 Tesla MRI (Ingenia 3 T Phillips). The sagittal MRI slices were set as true sagittal sequences. The cases were included only if there were no more than 3 days between the accident and the MRI. Retrospectively the tibial slope was measured by two independent orthopedic surgeons, a fellowship trained knee attending surgeon and a senior orthopedic resident in 116 MRI´s of patients with and in the same number of patients without an ACL rupture. The reviewers were blinded to the pathology and to each other´s results. The osseous as well as the cartilaginous slope was defined in the postero-latero-lateral (PLL) and postero-latero-central (PLC) segments of the tibial plateau using the 10-segment classification (Fig. 1). The PLL and PCL were determined by simultaneously checking the position in the axial images. In both segments, the osseous as well as the cartilaginous slope was measured. Intra- and inter-observer reliabilities were assessed. The measurements were repeated at an interval of 3 months with a random order of the studied knees.

If a posterolateral tibial plateau fracture occurred in the ACL rupture group, also known as an “apple bite fracture”, it was classified according to Menzdorf et al. [21].

**Fig. 2:**
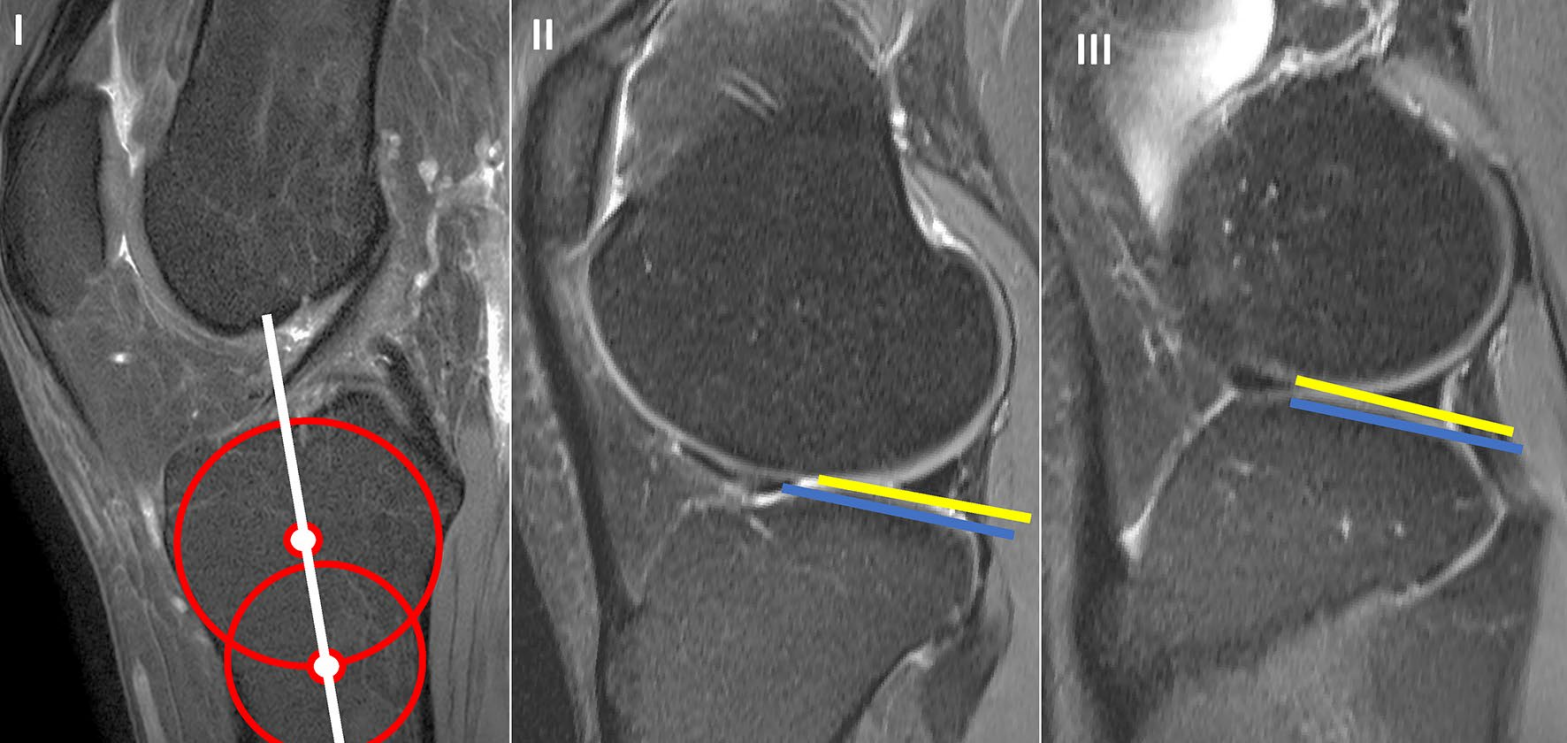
Measurement of tibial slope in the PLC and PLL segment according to Hudek et al. in a 31-year-old healthy woman. It shows the determination of the tibial shaft axis. The white line represents the tibial shaft axis and is defined by the centers of the two red circles. The proximal red circle is bounded by the anterior and posterior corticalis and the tibial plateau. The distal red circle is bounded by the anterior and posterior corticalis and the center of the circle lies on the circumference of the proximal circle; II sagittal MRI in the center of the PLC segment, the yellow line is the tangent representing the cartilaginous slope and the blue tangent represents the bony slope; III sagittal MRI in the center of the PLL, again the yellow line represents the tangent presenting the cartilaginous slope and the blue one the bony slope

Lateral tibial slope (LTS) were measured on sagittal MRI sequences according to the method described by Hudek et al. [22] (Fig. 2). This method has been reported to be the most repeatable method to measure sagittal tibial slopes on MRI and is independent of proximal tibial length [23]. First, the proximal anatomical axis of the tibia was defined on the central sagittal slice in which the attachment of the PCL and the intercondylar eminence were visualized, and both the anterior and posterior tibial cortices appeared in a concave shape. Within this slice, two circles were placed into the proximal tibia. The proximal circle was fit within the proximal, anterior, and posterior cortical borders. The distal circle was fit within the anterior and posterior cortices with the center of the circle positioned on the perimeter of the proximal circle. A line drawn through the centers of both circles defined the proximal anatomical axis of the tibia. Next, the sagittal slice showing the center of the PLL and PLC segment of the lateral plateau were identified. In the PLL as well as in the PLC a tangent to the uppermost even part between the superior anterior and posterior cortices was drawn. For the cartilaginous slope, the tangent was applied to the cartilage layer seen on MRI. The PLL slope, as also the PLC slope, was defined as the angle between the orthogonal to the proximal anatomic axis of the tibia and the tangent. All measurements were made in digital at our PACS system.

After positive ethics vote of the local ethics committee the mentioned study was performed.

### Statistical analysis

The intraclass correlation coefficients (ICCs) and associated 95% confidence intervals (CIs) of the ICCs quantified inter- and intraobserver reliability. ICC values were interpreted as follows: ICC = 1, perfect agreement; 0.81–0.99, excellent agreement; 0.61–0.80, substantial agreement; 0.41–0.60, moderate agreement; 0.21–0.40, fair agreement; 0.00–0.20, poor agreement [24]. A Shapiro–Wilk normality test and Kolmogorov–Smirnov test were performed to determine if the data were normally distributed. Pearson and Spearman coefficient were used for correlation of parametric and nonparametric data, respectively. Comparison of binomial data was performed using Fisher’s exact test. Comparison of two paired groups with parametric and nonparametric data was performed using paired *T* test and Wilcoxon signed rank test, respectively. Comparison of two unpaired groups with parametric and nonparametric data was performed using independent *T* test and Mann–Whitney *U* test, respectively. The level of significance for all tests was set at *P* ≤ 0.05. All data were analyzed using IBM SPSS Statistics version 26.0 (IBM, Armonk, NY).

## Results

There were 55 males and 61 females in the group with intact ACL and 70 males and 46 females in the group with a ruptured ACL. There was no significant difference between the two groups in terms of gender distribution (*P* 0.064). The average age in both groups was in the early thirties. However, the group with intact ACL was significantly older with an age of 33.3 ± 11.7 in comparison to the group with a ruptured ACL (30.6 ± 9.2, *P* 0.048) (Table 1). Bony PLL slope was found to be significantly higher in the group with torn ACL than in the group with intact ACL (9.8° ± 4.8° vs. 5.8° ± 4.8°, *P* < 0.001). Cartilaginous slope in the PLL segment also showed significantly higher values in the group with a ruptured ACL (10.4° ± 4.7° vs. 6.7° ± 4.8°, *P* < 0.001). In the PLC segment, both the bony (10.3° ± 4.8° vs. 6.6° ± 5.0°, *P* < 0.001) and cartilaginous (12.08° ± 4.3° vs. 9.4° ± 5.7°, *P* < 0.001) slopes were also significantly higher (Table 1).

**Table 1:** Demographics and radiographic assessment of slope in patients with intact (*N* = 116) vs. ruptured (*N* = 116) anterior crucial ligament

Parameter	Patients with intact ACL	Patients with ruptured ACL	*P* value
Gender			0.064^†^
Male	55	70	
Female	61	46	
Age (years)	33.3 ± 11.7	30.6 ± 9.2	0.048^‡^
Bony slope (PLL-segment) (°)	5.8 ± 4.8	9.8 ± 4.8	< 0.001^‡^
Cartilaginous slope (PLL segment) (°)	6.7 ± 4.8	10.4 ± 4.7	< 0.001^‡^
Bony slope (PLC segment) (°)	6.6 ± 5.0	10.3 ± 4.8	< 0.001^§^
Cartilaginous slope (PLC segment) (°)	9.4 ± 5.7	12.8 ± 4.3	< 0.001^§^

*ACL* anterior crucial ligament

^†^Using Fisher’s exact test

^‡^Using Mann–Whitney *U* test

^§^Using independent *T* test

There is also a significant difference between the cartilaginous and bony slope within the ACL ruptured and ACL intact group. However, there is a strong correlation between the cartilaginous and bony slope in the affected segment in all cases (Table 2).

**Table 2:** Radiographic assessment of bony vs. cartilage slope

	Bony slope (°)	Cartilaginous slope (º)	*P* value^a^	Correlation coefficient	*P* value^b^
Patients with intact ACL					
PLL segment	5.8 ± 4.8	6.7 ± 4.8	< 0.001^†^	0.886^‡^	< 0.001
PLC segment	6.6 ± 5.0	9.4 ± 5.7	< 0.001^§^	0.819^&^	< 0.001
Patients with ruptured ACL					
PLL segment	9.8 ± 4.8	10.4 ± 4.7	0.005^†^	0.900^‡^	< 0.001
PLC segment	10.3 ± 4.8	12.8 ± 4.3	< 0.001^§^	0.802^&^	< 0.001

*ACL* anterior crucial ligament

^†^Using Wilcoxon signed rank test

^‡^Spearman correlation coefficient

^§^Using paired *T* test

^&^Pearson correlation coefficient

^a^*P* value regarding the difference bony vs. cartilaginous slope

^b^*P* value regarding correlation coefficient

Of the 116 patients with an ACL rupture, 105 showed a posterolateral bone bruise with impression/fracture. According to Menzdorf et al. 88 of them had a 1a, four a 1b, three a 1c nine a 2a and one a 2b. No statistically significant difference in bony and cartilaginous slope was found in the PLL and PCL in the ACL ruptured cohort between those with a significant posterolateral impression/fracture and those without (Table 3).

**Table 3:** Demographics and radiographic assessment of slope in patients with (*N* = 105) vs. without (*N* = 11) posterolateral fracture in 116 patients with a ruptured anterior crucial ligament

Parameter	Patients with posterolateral fracture	Patients without posterolateral fracture	*P* value
Gender			1.000^†^
Male	63	7	
Female	42	4	
Bony slope (PLL-segment) (°)	9.8 ± 5.0	9.7 ± 1.7	0.823^‡^
Cartilaginous slope (PLL segment) (°)	10.4 ± 4.9	10.0 ± 2.6	0.797^‡^
Bony slope (PLC segment) (°)	10.5 ± 4.8	8.2 ± 3.6	0.134^§^
Cartilaginous slope (PLC segment) (°)	12.7 ± 4.3	12.8 ± 4.4	0.968^§^

^†^Using Fisher’s exact test

^‡^Using Mann–Whitney *U* test

^§^Using independent *T* test

However, a significant difference in bony (*P* = 0.001) and cartilaginous (*P* = 0.005) PLL slope as well as in cartilaginous PLC (*P* = 0.032) slope was observed between the 11 patients with ACL rupture without posterolateral impression/fracture and the healthy group (patients without ACL rupture and without posterolateral fracture).

No statistical significance could be demonstrated in the bony slope of the PLC segment between these groups (Table 4).

**Table 4:** Demographics and radiographic assessment of slope in patients with an ACL rupture and without and posterolateral fracture (*N* = 11) vs. without ACL rupture and without posterolateral fracture (*N* = 116)

Parameter	Patients without ACL rupture and without posterolateral fracture	Patients with ACL rupture and without posterolateral fracture	*P* value
Gender			1.000^†^
Male	55	7	
Female	61	4	
Bony slope (PLL-segment) (°)	5.8 ± 4.8	9.7 ± 1.7	0.001^‡^
Cartilaginous slope (PLL segment) (°)	6.7 ± 4.8	10.0 ± 2.6	0.005^‡^
Bony slope (PLC segment) (°)	6.6 ± 5.0	8.2 ± 3.6	0.186^§^
Cartilaginous slope (PLC segment) (°)	9.4 ± 5.7	12.8 ± 4.4	0.032^§^

^†^Using Fisher’s exact test

^‡^Using Mann–Whitney *U* test

^§^Using independent *T* test

The inter- and intraobserver reliability showed excellent results according to the benchmark definitions of Fleiss [24]. Thereby the interobserver reliability shows 87.0–98.2% for the bony or cartilaginous slope in the respective segment. For intraobserver reliability, the results from 85.8% up to 99.6% were observed (Table 5).

**Table 5:** Interobserver and intraobserver reliability with interrater correlation coefficient for slope measurements in patients with and without ruptured anterior crucial ligament (*N* = 116)

ACL intact		ACL ruptured	
Interobserver reliability	Intraobserver reliability^a^	Interobserver reliability	Intraobserver reliability^a^
Bony slope (postero-latero-lateral segment)			
0.932^†^ (*P* < 0.001^†^)	0.996^†^ (*P* < 0.001^†^)	0.982^†^ (*P* < 0.001^†^)	0.935^†^ (*P* < 0.001^†^)
95% CI 0.845–0.970	95% CI 0.990–0.998	95% CI 0.958–0.992	95% CI 0.853–0.971
Excellent^‡^	Excellent^‡^	Excellent^‡^	Excellent^‡^
Cartilage slope (postero-latero-lateral segment)			
0.870^†^ (*P* < 0.001^†^)	0.993^†^ (*P* < 0.001^†^)	0.979^†^ (*P* < 0.001^†^)	0.921^†^ (*P* < 0.001^†^)
95% CI 0.705–0.943	95% CI 0.984–0.997	95% CI 0.951–0.991	95% CI 0.821–0.965
Excellent^‡^	Excellent^‡^	Excellent^‡^	Excellent^‡^
Bony slope (postero-latero-central segment)			
0.961^†^ (*P* < 0.001^†^)	0.996^†^ (*P* < 0.001^†^)	0.979^†^ (*P* < 0.001^†^)	0.948^†^ (*P* < 0.001^†^)
95% CI 0.911–0.983	95% CI 0.991–0.998	95% CI 0.953–0.991	95% CI 0.881–0.977
Excellent^‡^	Excellent^‡^	Excellent^‡^	Excellent^‡^
Cartilage slope (postero-latero-central segment)			
0.947^†^ (*P* < 0.001^†^)	0.993^†^ (*P* < 0.001^†^)	0.963^†^ (*P* < 0.001^†^)	0.858^†^ (*P* < 0.001^†^)
95% CI 0.879–0.977	95% CI 0.984–0.997	95% CI 0.916–0.984	95% CI 0.677–0.937
Excellent^‡^	Excellent^‡^	Excellent^‡^	Excellent^‡^

95% CI 95% confidence interval

^†^*F* test significance

^‡^Agreement assessment according to the benchmark definitions of Fleiss [24]

^a^Fellowship trained knee attending surgeon

## Discussion

Our results show a physiological value of 5.8° ± 4.8° for the bony and 6.7° ± 4.8° for the cartilaginous slope in the PLL segment and for the PLC segment of 6.6° ± 5.0° bony and 9.4° ± 5° cartilaginous. A steeper slope could be detected in ACL ruptured patients.

Hudek et al. showed a medial slope of 4.8° ± 0.5° and a lateral slope of 5.0° ± 3.6° in 100 healthy knee joints. Our results indicate that the bony slope of the PLC and PLL is about 0.8°–1.6° higher than measured in the center of the lateral plateau.

In a comparison of slope measurements on X-ray, CT, and MRI, Utzschneider et al. found an average medial tibial slope of 9.3° ± 2.2° and a lateral slope of 10.0° ± 2.2° on MRI in healthy knee joints [25]. However, these values have been measured with a different measurement method. Wordeman et al. were able to show that the slope is significantly influenced by the method of measurement and that there is a high variability between the measured values [26].

To our knowledge, there is no study that has determined the bony and cartilaginous slope in the area of the PLC and the PLL in healthy subjects and compared it with a group of patients with ruptured ACL.

However, there are studies that attribute particular importance to the lateral tibial plateau in relation to injuries of the knee joint [27, 28]. Stijak et al. compared a healthy control group with an ACL ruptured group and found a significant increase in lateral tibial slope in the ACL ruptured group. Thereby, there was not only a greater lateral slope between the healthy and torn ACL groups, but also a greater lateral slope than the medial slope in the torn ACL group [27]. This is consistent with our findings that compared with the healthy control group, the slope in both segments was significantly increased in the group with torn ACL.

Some authors have shown that the soft tissue slope consisting of cartilage and meniscus is lower than the bony slope [29, 30]. Nevertheless, measuring the soft tissue inclination in the area of the posterolateral quadrant of the tibial plateau seems to have major downsides. Under load, loss of the meniscal root could convert the effective functional slope from the meniscal slope to the bony slope. In addition, MRI is performed in the supine position and weight-bearing may affect the meniscal height both anteriorly and posteriorly and thus alter the measured meniscal slope [29, 30].

Furthermore, measuring the isolated soft tissue inclination with meniscus in the area of the posterolateral quadrant of the tibial plateau is technically not possible. Using our method, a second reference point is missing to generate a straight line, since the anterior horn of the meniscus lies within the anterolateral quadrant and not in the posterolateral quadrant. Measurement in the posterolateral quadrant would mainly represent the angle of the posterior meniscus. Therefore, the cartilaginous slope was determined as described. In our measurements, this slope is significantly higher in the PLL and PLC than the bony slope. As with the bony slope, to our knowledge there is no data on the cartilaginous slope in the posterolateral quadrant of the tibial plateau.

The analysis showed a strong correlation between the cartilaginous and the bony slope so that only one has to be considered in the measurement and therapy decision. The work of Khan et al. found a patient group with similar age (32.1 ± 7.5 years) and a correlation between the increase in lateral tibial plateau chondropathy and slope in the lateral tibial plateau [15]. However, the cartilage thickness was not measured at defined points. An assessment of the entire cartilage surface of the lateral plateau was performed and finally classified into 4 degrees of severity [31]. Thus, in our opinion, the study by Khan et al. only allows the statement that a steeper slope is found in patients with increasing osteoarthritis. A statement as to whether increasing osteoarthritis leads to a steeper slope cannot be made due to the study design. Furthermore Cai et al. found in a longitudinal cohort study a significant trend that older participants lost more tibial cartilage volume at both medial and lateral compartments [32]. Taking this into account, it can be postulated that, depending on the localization of the osteoarthritis, an influence on the tibial slope may also occur. A prospective longitudinal study would be necessary for this purpose.

In our study, there was no significant difference in the slope between cases with a posterolateral bone bruising or fracture and those without. Nevertheless, it should be noted that 1a and 2a fractures were predominant in our population. These show only bone bruising with a small impaction or dislocation (< 2 mm) and affect only a small part of the segment. That is why they seem to have little effect on the slope, which supports the previous decision to treat these conservatively [21].

Even though only in bony PLL, cartilaginous PLL and cartilaginous PLC a significant difference was found between the healthy and the “no fracture” ACL ruptured group. Also, the bony PLC slope was steeper in the collective with torn ACL without tibial fracture compared to the healthy group. In addition, we showed that the bony and cartilaginous slopes are correlated. Therefore, the lack of statistical significance in the bony PLC slope is due to the limited group size of 11 patients without a posterolateral bone bruising or fracture. This implies that the tibial slope already plays an important role in the development of anterior cruciate ligament injury. This is consistent with the results of Stijak et al. who found a steeper lateral tibial slope in ACL ruptured patients [27]. In further studies, a correlation between a steeper slope as measured in radiographs and ACL injuries in women (with noncontact injury mechanism) as well as children compered to nonruptured ACL group has already been established [33, 34]. In addition, our results support the recommendation of Winkler et al. to assess the tibial slope in both re-ruptured ACL and primary ACL ruptures [35]. The results of this paper indicate that it might not only be tibial slope in general but especially the tibial slope of the posterolateral quadrant, which could be a risk factor for an ACL injury. Our results at least indicate that the tibial slope should be considered differentially. However, whether this has a therapeutic consequence is still controversial [24].

### Limitations

The limitations of the study are the retrospective single center design as well as the unequal study size with respect to the posterolateral bone bruise and impression fracture. Although for the normal values of the PLL and PLC slope the study size according to the power analysis is large enough for a statistically significant statement.

## Conclusion

In conclusion, our study gives first normal values: the bony slope of the PLL is 5.8° ± 4.8° and of the PLC is 6.6° ± 5.0° and the cartilaginous slope is 6.7° ± 4.8° (PLL) or 9.4° ± 5.7° (PLC) in healthy individuals measured in MRI. In addition, the bony slope and the cartilaginous slope in this area are different, but not independent.

Considering our findings, it can be stated that in our patient collective with anterior cruciate ligament injury there is a significantly steeper slope in the posterolateral quadrant compared to the healthy group. Thus, there might be a correlation between a steeper slope and the occurrence of an ACL injury.

## Abbreviations

ACL: Anterior cruciate ligament
ATT: Anterior tibial translation
PLC: Posterolateral central segment
PLL: Posterolateral lateral segment
LTS: Lateral tibial slope
ICCs: Intraclass correlation coefficients
CIs: Confidence intervals

## References

[1] Kim SY, Spritzer CE, Utturkar GM, Toth AP, Garrett WE, DeFrate LE (2015). Knee kinematics during noncontact anterior cruciate ligament injury as determined from bone bruise location. Am J Sports Med.

[2] Horstmann H, Petri M, Tegtbur U, Felmet G, Krettek C, Jagodzinski M (2021). Quadriceps and hamstring tendon autografts in ACL reconstruction yield comparably good results in a prospective, randomized controlled trial. Arch Orthop Trauma Surg.

[3] Korthaus A, Warncke M, Pagenstert G, Krause M, Frosch KH, Kolb JP (2021). Lateral femoral notch sign and posterolateral tibial plateau fractures and their associated injuries in the setting of an anterior cruciate ligament rupture. Arch Orthop Trauma Surg.

[4] Bernholt DL, DePhillipo NN, Crawford MD, Aman ZS, Grantham WJ, LaPrade RF (2020). Incidence of displaced posterolateral tibial plateau and lateral femoral condyle impaction fractures in the setting of primary anterior cruciate ligament tear. Am J Sports Med.

[5] Marouane H, Shirazi-Adl A, Hashemi J (2015). Quantification of the role of tibial posterior slope in knee joint mechanics and ACL force in simulated gait. J Biomech.

[6] Giffin JR, Vogrin TM, Zantop T, Woo SL, Harner CD (2004). Effects of increasing tibial slope on the biomechanics of the knee. Am J Sports Med.

[7] Feucht MJ, Mauro CS, Brucker PU, Imhoff AB, Hinterwimmer S (2013). The role of the tibial slope in sustaining and treating anterior cruciate ligament injuries. Knee Surg Sports Traumatol Arthrosc.

[8] Butler DL, Noyes FR, Grood ES (1980). Ligamentous restraints to anterior–posterior drawer in the human knee. A biomechanical study. J Bone Jt Surg Am.

[9] Imhoff FB, Mehl J, Comer BJ, Obopilwe E, Cote MP, Feucht MJ (2019). Slope-reducing tibial osteotomy decreases ACL-graft forces and anterior tibial translation under axial load. Knee Surg Sports Traumatol Arthrosc.

[10] Grassi A, Macchiarola L, Urrizola Barrientos F, Zicaro JP, Costa Paz M, Adravanti P (2019). Steep posterior tibial slope, anterior tibial subluxation, deep posterior lateral femoral condyle, and meniscal deficiency are common findings in multiple anterior cruciate ligament failures: an MRI case–control study. Am J Sports Med.

[11] Schneider A, Arias C, Bankhead C, Gaillard R, Lustig S, Servien E (2020). Greater medial tibial slope is associated with increased anterior tibial translation in females with an ACL-deficient knee. Knee Surg Sports Traumatol Arthrosc.

[12] Herbort M (2020) The “Bankart knee”: biomechanical consequences of a posterolateral tibia plateau impression fracture as concomitant injury of ACL rupture. ACL study group, Kitzbuehel. Kitzbuehel

[13] Kawashima I, Tsukahara T, Sakai T, Kawai R, Ishizuka S, Hiraiwa H (2021). Delayed anterior cruciate ligament reconstruction increases the incidence of medial meniscal bucket handle tears and medial compartment chondral injuries in patients aged 40 years and older. Arch Orthop Trauma Surg.

[14] Akoto R, Alm L, Drenck TC, Frings J, Krause M, Frosch K-H (2020). Slope-correction osteotomy with lateral extra-articular tenodesis and revision anterior cruciate ligament reconstruction is highly effective in treating high-grade anterior knee laxity. Am J Sports Med.

[15] Alm L, Drenck TC, Frosch KH, Akoto R (2020). Lateral extra-articular tenodesis in patients with revision anterior cruciate ligament (ACL) reconstruction and high-grade anterior knee instability. Knee.

[16] Kolbe R, Schmidt-Hebbel A, Forkel P, Pogorzelski J, Imhoff AB, Feucht MJ (2019). Steep lateral tibial slope and lateral-to-medial slope asymmetry are risk factors for concomitant posterolateral meniscus root tears in anterior cruciate ligament injuries. Knee Surg Sports Traumatol Arthrosc.

[17] Sabzevari S, Rahnemai-Azar AA, Shaikh HS, Arner JW, Irrgang JJ, Fu FH (2017). Increased lateral tibial posterior slope is related to tibial tunnel widening after primary ACL reconstruction. Knee Surg Sports Traumatol Arthrosc.

[18] Ackermann C, Frings J, Alm L, Frosch KH (2019). Arthroscopic controlled closed reduction and percutaneous fixation of posterolateral tibia plateau impression fractures. Arthrosc Tech.

[19] Amerinatanzi A, Summers RK, Ahmadi K, Goel VK, Hewett TE, Nyman E (2017). Automated measurement of patient-specific tibial slopes from MRI. Bioengineering (Basel).

[20] Krause M, Preiss A, Müller G, Madert J, Fehske K, Neumann MV (2016). Intra-articular tibial plateau fracture characteristics according to the “Ten segment classification”. Injury.

[21] Menzdorf L, Drenck T, Akoto R, Hartel M, Krause M, Guttowski D (2020). Clinical results after surgical treatment of posterolateral tibial plateau fractures (“apple bite fracture”) in combination with ACL injuries. Eur J Trauma Emerg Surg.

[22] Hudek R, Schmutz S, Regenfelder F, Fuchs B, Koch PP (2009). Novel measurement technique of the tibial slope on conventional MRI. Clin Orthop Relat Res.

[23] Lipps DB, Wilson AM, Ashton-Miller JA, Wojtys EM (2012). Evaluation of different methods for measuring lateral tibial slope using magnetic resonance imaging. Am J Sports Med.

[24] Fleiss JL, Levin B, Paik MC (1981). The measurement of interrater agreement. Stat Methods Rates Proportions.

[25] Utzschneider S, Goettinger M, Weber P, Horng A, Glaser C, Jansson V (2011). Development and validation of a new method for the radiologic measurement of the tibial slope. Knee Surg Sports Traumatol Arthrosc.

[26] Wordeman SC, Quatman CE, Kaeding CC, Hewett TE (2012). In vivo evidence for tibial plateau slope as a risk factor for anterior cruciate ligament injury: a systematic review and meta-analysis. Am J Sports Med.

[27] Stijak L, Herzog RF, Schai P (2008). Is there an influence of the tibial slope of the lateral condyle on the ACL lesion?. Knee Surg Sports Traumatol Arthrosc.

[28] Jenny JY, Rapp E, Kehr P (1997). Proximal tibial meniscal slope: a comparison with the bone slope. Rev Chir Orthop Reparatrice Appar Mot.

[29] Cinotti G, Sessa P, Ragusa G, Ripani FR, Postacchini R, Masciangelo R (2013). Influence of cartilage and menisci on the sagittal slope of the tibial plateaus. Clin Anat.

[30] Elmansori A, Lording T, Dumas R, Elmajri K, Neyret P, Lustig S (2017). Proximal tibial bony and meniscal slopes are higher in ACL injured subjects than controls: a comparative MRI study. Knee Surg Sports Traumatol Arthrosc.

[31] Khan N, Shepel M, Leswick DA, Obaid H (2014). Increasing lateral tibial slope: is there an association with articular cartilage changes in the knee?. Skelet Radiol.

[32] Cai G, Jiang M, Cicuttini F, Jones G (2019). Association of age, sex and BMI with the rate of change in tibial cartilage volume: a 10.7-year longitudinal cohort study. Arthritis Res Ther.

[33] Vyas S, van Eck CF, Vyas N, Fu FH, Otsuka NY (2011). Increased medial tibial slope in teenage pediatric population with open physes and anterior cruciate ligament injuries. Knee Surg Sports Traumatol Arthrosc.

[34] Hohmann E, Bryant A, Reaburn P, Tetsworth K (2011). Is there a correlation between posterior tibial slope and non-contact anterior cruciate ligament injuries?. Knee Surg Sports Traumatol Arthrosc.

[35] Winkler PW, Godshaw BM, Karlsson J, Getgood AMJ, Musahl V (2021). Posterior tibial slope: the fingerprint of the tibial bone. Knee Surg Sports Traumatol Arthrosc.

